# Promotion of mental health in young adults via mobile phone app: study protocol of the ECoWeB (emotional competence for well-being in Young adults) cohort multiple randomised trials

**DOI:** 10.1186/s12888-020-02857-w

**Published:** 2020-09-22

**Authors:** A. Newbold, F. C. Warren, R. S. Taylor, C. Hulme, S. Burnett, B. Aas, C. Botella, F. Burkhardt, T. Ehring, J. R. J. Fontaine, M. Frost, A. Garcia-Palacios, E. Greimel, C. Hoessle, A. Hovasapian, VEI Huyghe, J. Lochner, G. Molinari, R. Pekrun, B. Platt, T. Rosenkranz, K. R. Scherer, K. Schlegel, G. Schulte-Korne, C. Suso, V. Voigt, E. R. Watkins

**Affiliations:** 1 Mood Disorders Centre, School of Psychology, Sir Henry Wellcome Building for Mood Disorders Research, University of Exeter, Exeter, EX4 4LN UK; 2 College of Medicine and Health, University of Exeter, Exeter, UK; 3 MRC/CSO Social and Public Health Sciences Unit & Robertson Centre for Biostatistics, Institute of Health and Well Being, University of Glasgow, Glasgow, UK; 4 Department of Child and Adolescent Psychiatry, Psychosomatics and Psychotherapy, University Hospital, LMU, Munich, Germany; 5 Universitat Jaume I, Castelló de la Plana, Spain; 6 CIBER Fisiopatología Obesidad y Nutrición (CIBERObn), Instituto Salud Carlos III, Madrid, Spain; 7 audEERING, Munich, Germany; 8 Department of Psychology, LMU Munich, Munich, Germany; 9 Department of Work, Organization and Society, Ghent University, Ghent, Belgium; 10 Monsenso ApS, Copenhagen, Denmark; 11 Department of Psychology, University of Essex, UK, and Institute for Positive Psychology and Education, Australian Catholic University, Sydney, Australia; 12 University of Geneva, Geneva, Switzerland; 13 University of Bern, Bern, Switzerland

**Keywords:** Depression, Well-being, Personalization, Young people, Mobile-health prevention, Randomised controlled trial, Emotional competence, Rumination

## Abstract

**Background:**

Promoting well-being and preventing poor mental health in young people is a major global priority. Building emotional competence (EC) skills via a mobile app may be an effective, scalable and acceptable way to do this. However, few large-scale controlled trials have examined the efficacy of mobile apps in promoting mental health in young people; none have tailored the app to individual profiles.

**Method/design:**

The Emotional Competence for Well-Being in Young Adults cohort multiple randomised controlled trial (cmRCT) involves a longitudinal prospective cohort to examine well-being, mental health and EC in 16–22 year olds across 12 months. Within the cohort, eligible participants are entered to either the PREVENT trial (if selected EC scores at baseline within worst-performing quartile) or to the PROMOTE trial (if selected EC scores not within worst-performing quartile). In both trials, participants are randomised (i) to continue with usual practice, repeated assessments and a self-monitoring app; (ii) to additionally receive generic cognitive-behavioural therapy self-help in app; (iii) to additionally receive personalised EC self-help in app. In total, 2142 participants aged 16 to 22 years, with no current or past history of major depression, bipolar disorder or psychosis will be recruited across UK, Germany, Spain, and Belgium. Assessments take place at baseline (pre-randomisation), 1, 3 and 12 months post-randomisation. Primary endpoint and outcome for PREVENT is level of depression symptoms on the Patient Health Questionnaire-9 at 3 months; primary endpoint and outcome for PROMOTE is emotional well-being assessed on the Warwick-Edinburgh Mental Wellbeing Scale at 3 months. Depressive symptoms, anxiety, well-being, health-related quality of life, functioning and cost-effectiveness are secondary outcomes. Compliance, adverse events and potentially mediating variables will be carefully monitored.

**Conclusions:**

The trial aims to provide a better understanding of the causal role of learning EC skills using interventions delivered via mobile phone apps with respect to promoting well-being and preventing poor mental health in young people. This knowledge will be used to develop and disseminate innovative evidence-based, feasible, and effective Mobile-health public health strategies for preventing poor mental health and promoting well-being.

**Trial registration:**

ClinicalTrials.gov (www.clinicaltrials.org). Number of identification: NCT04148508 November 2019.

## Background

There is growing global concern about the high and steadily increasing rates of poor mental health in young people and of the early onset of mental disorders such as anxiety and depression [1]. Such poor mental health during this key formative period severely affects the future life chances of young people, with significant long-term impact on future health, education, employment and social outcomes [1–4]. The incidence of anxiety and depression each markedly increase from mid-adolescence through to young adulthood, peaking during this period [2]. As a consequence, there has been a call for urgent improvement in primary prevention of poor mental health, and for improvement in promotion of mental well-being [4, 5].

Although there are already evidence-based effective primary prevention interventions for common mental health disorders including Social-Emotional Learning (SEL) programmes [6] and cognitive-behavioural approaches [7], systematic reviews suggest that effect sizes are relatively small and there is scope to increase intervention efficacy [7–10]. Furthermore, most current evidence-based interventions require considerable person-hours from professionals because of the involvement of a relevant workforce such as teachers and therapists. Ideally, we need mental health promotion and prevention approaches that do not require considerable person-hours and that are highly scalable to a population level, that can be delivered as a public health approach, and that can increase treatment efficacy.

To tackle these key challenges, the Assessing and Enhancing Emotional Competence for Well-being in the Young (ECoWeB) project innovatively integrates three approaches: basing interventions on a model of normal emotional functioning; personalising intervention based on baseline scores; and the use of smartphone app delivery. First, rather than basing mental health promotion and mental ill-health prevention on traditional clinical disease models of psychopathology, we adopt an approach based on an established theoretical model of normal emotional functioning – the Component Process Model of Emotion (CPM) [11–13]. This model proposes that individuals vary in their abilities across different areas of Emotional Competence (EC), including: (i) accurate and functional appraisals of emotional situations and of the individual’s ability to cope with these situations, which determines whether an individual experiences the emotion appropriate to a situation (Emotion Production); (ii) abilities to perceive and understand emotions in themselves and others (Emotion Knowledge and Perception); (iii) and the use of more adaptive versus less adaptive strategies to manage and regulate emotions (Emotion Regulation). The model hypothesizes that good EC functioning contributes to reduced anxiety and depression, and improved mental well-being. Considerable correlational and prospective data is consistent with this hypothesis [14–19]. Targeting hypothesized underlying mechanisms common across all individuals no matter their symptomatology has the potential to enhance intervention efficacy and is particularly well-suited to the promotion of well-being and prevention of poor mental health at the population-level. As such, the ECoWeB project will test the efficacy of a self-help intervention focused on building EC to promote well-being and prevent poor mental health in young people.

Second, ECoWeB adopts a personalisation approach in which individuals in the experimental intervention are offered specific self-help psychoeducation, strategies and training matched to their EC profile, on the hypothesis that a tailored intervention will be more acceptable and efficacious than a generic intervention. This approach has not yet been evaluated in well-being and mental health promotion/prevention research despite recent arguments for predictive, personalised, preventive and participatory medicine focused on individual well-being [20].

Third, ECoWeB will investigate the delivery of the self-help intervention through a mobile app. The use of mobile apps (sometimes called Behavioural Intervention Technologies, BITs) [21] has a number of potential advantages: (i) scalability–Mobile-health (m-health) technologies are highly scalable, allowing very good coverage and reach; they are widely accessible; (ii) non-consumable–they enable repeated use by nearly unlimited people simultaneously; (iii) convenience – they can be used anytime, anywhere; (iv) acceptability – mobile apps are highly used by young people, with the majority of young people using smart phones [22]. In addition, mobile apps can help to integrate behavioural changes into daily life: the app is always on hand via the smartphone, making it well-suited for changing habits. Despite huge increase in the numbers of m-health apps (> 10 k) [23], only a very small minority have been based on robust science, utilised established treatment principles and been rigorously tested with respect to safety and efficacy in robust well-powered randomised controlled trials (RCTs) [24–27]. There is emerging evidence for m-health apps as efficacious treatment interventions for anxiety and depression [28, 29], although the average sample size is still under 100 participants per trial arm, and few trials have examined well-being promotion and prevention of poor mental health specifically in young people. To our knowledge, ECoWeB will be the first fully powered definitive trial of an m-health app for mental health promotion in young people.

A further issue within the field of mental health promotion and prevention of poor mental health is the definition of the target population of the intervention. Population-oriented public health approaches are often available to the general eligible population without specific targeting (i.e., a universal intervention). However, there is some evidence that preventive interventions that are selective and target specific higher-risk groups within the wider eligible population, such as those with subsyndromal symptoms or indicative risk factors, may be more efficacious [8, 10]. Because this remains unresolved and has considerable implications for future implementation, ECoWeB will include two RCTs: one selecting individuals with indicative elevated risk for future poor mental health based on baseline EC profile (a targeted high-risk population: ECoWeB-PREVENT), and the other including participants without such indicative elevated risk (a low-risk population: ECoWeB-PROMOTE), within a single cohort multiple randomised controlled trial (cmRCT) design [30]. In a cmRCT, individuals meeting relevant criteria are recruited into a large-scale prospective cohort and their outcomes are regularly monitored across the prospective period. In addition to consenting to be assessed over the follow-up period, participants also consent to being offered additional intervention if eligible. For each potential RCT in the cohort, information from the cohort is used to identify all eligible participants and then some eligible participants are randomly selected and offered the additional intervention.

We stress that the two trials share the same recruitment procedure, interventions, set of outcome measures and designs and most eligibility criteria. As the funding remit for ECoWeB was for promotion of mental well-being and primary prevention of mental disorders, both trials exclude those with a history of current or past major depression or a diagnosis of bipolar disorder or psychosis: individuals passing these criteria are eligible for either PREVENT or PROMOTE depending on their EC profile. The key differences between the trials are the eligibility criteria with respect to EC profile for each trial and as a consequence the primary outcome measure. The ECoWeB-PREVENT trial will recruit participants who have a hypothesized elevated risk of poor mental health based on an EC profile in which they score in the worst quartile on at least one EC component, such as a greater tendency towards well-established unhelpful emotional regulation strategies such as rumination [31–33] or making negative appraisals [34]. For this group, there is increased likelihood of worsening mental health over 12 months and we choose depression symptoms (Patient Health Questionnaire-9) as our primary outcome as potentially the most sensitive and important index of poor mental health. ECoWeB-PREVENT therefore tests whether provision of personalised digital EC self-help can prevent the onset and increase of depression symptoms relative to generic digital CBT self-help and the usual practice self-monitoring control.

The ECoWeB-PROMOTE trial will recruit participants not showing elevated risk on their EC profile, that is, relatively well and high-functioning individuals, who do not score in the worst quartile for any of the EC components. For this group, there is lower likelihood of worsening mental health over 12 months. The sensible primary outcome is therefore increased mental well-being, indexed by the Warwick-Edinburgh Mental Wellbeing Scale. ECoWeB-PROMOTE therefore tests whether the provision of personalised digital EC self-help can improve well-being relative to generic digital CBT self-help and the usual practice self-monitoring control. Both trials share the same secondary outcome measures so that well-being promotion and prevention of poor mental health can be tested in both.

Because there are many different emotional competence skills that could potentially be assessed and trained, for initial feasibility and to test proof-of-principle for this approach, we identified at least one process to target with respect to each EC domain. Targets were therefore chosen that had been robustly implicated in mental health and well-being, and already had well-established validated assessment measures. These targets were also readily adaptable and proven interventions, albeit typically delivered through different media than apps, such as web-based therapy and classroom interventions. On this basis, the four target processes selected were appraisal biases in achievement contexts; interpretative biases in social contexts, each reflecting the Emotion Production component; rumination and worry reflecting proven dysfunctional strategies in Emotion Regulation; and emotional knowledge, understanding and recognition. Specifically, there are four components within the personalized digital EC self-help intervention, with each individual participant receiving their two lowest-ranked EC components based on their EC profile.

The achievement-focused appraisal self-help component builds on the control-value theory of achievement emotions [35] and aims to address lack of control, lack of value, and excessive negative value. To do so, it combines three established treatment approaches: (a) attributional retraining intended to build internal and controllable attributions for academic or work-related outcomes to increase perceived control, building on a video-based short intervention that has already had benefit in college students [36–38]; (b) mindset training to promote perceived control and growth mindsets, found to improve academic outcomes [39, 40] and which has been shown to reduce and prevent depression and anxiety in young people over 9 months after a single 30-min session [41–43]; (c) utility value self-help intended to increase the perceived value and usefulness of academic study and employment-related tasks [44, 45], as well as to reduce excessive concerns and fears of failure (excessive attainment value).

The social-focused appraisal self-help component builds on a model of youth depression which argues that negative appraisals may mediate the harmful effects of peer rejection on depressive symptoms [46]. Using an established Cognitive Bias Modification of Interpretations (CBM-I) paradigm, the Ambiguous Scenarios Task (AST), participants are trained over repeated trials to draw more positive interpretations of ambiguous social scenarios. Laboratory studies have demonstrated CBM-I to show medium to large effect sizes (ESs) in modifying negative interpretations in adults (g = 0.52 to 0.81) [47–49] and youth (g = 0.52 to 0.70) [50]. CBM-I has been shown to have modest benefits in reducing anxiety, and to a lesser extent depression in adults (g = 0.23) [47, 48] and youth with elevated symptoms (g = 0.17 to 0.34) [50], although the true clinical benefit has been questioned in some meta-analyses [47, 51, 52].

To target the Emotion Regulation EC component, ECoWeB adapts an existing proven intervention that targets a shift away from maladaptive worry and rumination, which are well-established risk factors for poor mental health [32, 33] to more adaptive problem-solving. This rumination self-help intervention builds on proven cognitive-behavioural therapy principles and includes identifying warning signs for worry, repeated practice to train out of unhelpful habits and build helpful habits, and the training of useful alternative strategies such as being more specific, relaxation, problem-solving and self-compassion [53]. This intervention has been proven to be effective in reducing and preventing depression and anxiety in face-to-face therapy [54–56] and in web-based interventions for young adults [57] including an entirely self-help variant [58].

The emotional knowledge and perception self-help component educates and enhances EC knowledge and perception through the provision of detailed psychoeducation and adapted assessment tasks in which immediate feedback helps users to build their skills. For example, training feedback on the Geneva Emotion Recognition Test which involves judging emotional expressions from video clips (GERT) [59, 60] has been found to improve performance on emotion recognition tests and increased co-operation with a stranger.

## Objective

The primary objective of the ECoWeB-PROMOTE trial is to examine the efficacy of personalized digital EC self-help relative to generic digital CBT self-help and a usual practice self-monitoring control to improve mental well-being at 3-month follow-up in young people with an EC profile without elevated hypothesized risk.

The primary objective of the ECoWeB-PREVENT trial is to examine the efficacy of personalized digital EC self-help relative to generic digital CBT self-help and a usual practice self-monitoring control to reduce poor mental health indexed by self-reported depression at 3-month follow-up in young people with an EC profile with elevated hypothesized risk.

For both trials, secondary objectives are to examine the efficacy of the interventions on secondary outcomes concerning mental well-being and mental health and associated costs, the maintenance of these effects at 12 month follow-up and to examine potential mediators and moderators of the beneficial effects (if any) of these m-health interventions.

## Methods

The study will be conducted and reported according to Consolidated Standards of Reporting Trials (CONSORT) [61, 62] and extensions for non-pharmacologic treatment interventions and multi-arm parallel-group randomised trials and CONSORT-EHEALTH for improving and standardising evaluation reports of Web-based and mobile health interventions [63].

### Study design

The trial design is of a cohort multiple randomised controlled trial (cmRCT) [30] involving two superiority parallel 3-arm randomised multicentre, multinational RCTs (ECoWeB-PREVENT; ECoWeB-PROMOTE). The arms are the same across both trials and all participants join the 12 month prospective longitudinal cohort.

Within ECoWeB, all participants recruited into the prospective cohort will be allocated into either the PREVENT or PROMOTE trial, and all are then randomised into one of three arms. The default arm within the cohort, that is, the control trial arm, involves completing web-based assessments at baseline and at 1-, 3- and 12-month follow-up, of well-being, mental health symptoms and EC functioning, and having access to a version of the app that supports self-monitoring of the participant’s emotions, plus any additional usual care a participant may receive external to the trial (henceforward, “usual practice self-monitoring control”). The active experimental arm involves the receipt of personalised EC self-help strategies within the app, in addition to the repeated measures and self-monitoring. In addition, there is an active control arm consisting of generic CBT self-help strategies within the app, in addition to the repeated measures and self-monitoring. The outcomes of participants randomly selected to each of the experimental and active control arms are compared with eligible participants randomly selected to remain in the usual practice self-monitoring control arm, and with each other.

There are multiple advantages to this cmRCT design. First, it effectively combines a prospective long-term longitudinal cohort design with a randomised trial. Random selection of some participants within each trial to additional intervention is equivalent to random allocation of all participants within each trial with respect to generating groups where all known and unknown prognostic factors are distributed evenly at baseline, enabling strong causal inference about the causal effects of each intervention. Nonetheless, retaining the default prospective cohort group provides information as to the natural history of well-being, emotions and mental health in this sample and allows us to examine the trajectory of well-being and symptoms over time and their relationship to EC.

Second, it potentially improves the efficiency and representativeness of sample recruitment as the study can be open widely to eligible young people and advertised as participating in a cohort study to learn about young people’s emotions as well as a clinical trial. Third, because individuals consent in advance to the option of having an intervention offered if eligible, we avoid individuals being knowingly allocated to a “lesser” usual care condition, potentially enhancing recruitment and retention.

Potential participants for either PROMOTE or PREVENT provide initial consent to complete screening measures to determine if they are eligible to participate in either of the two trials (ECoWeB-PROMOTE, EC profile not showing hypothesized elevated risk for future poor mental health; ECoWeB-PREVENT, EC profile showing hypothesized elevated risk for future poor mental health). Any potential participants who are found not to be eligible for either trial are automatically signposted to other sources of support. Once trial eligibility has been determined and consent to participate in the trial has been obtained, participants are individually selected at random (in a 1:1:1 ratio) to be offered self-help components within a mobile phone app (either personalised Emotional Competence self-help or general CBT self-help) or not to be contacted about the offered self-help and to continue with the app for self-monitoring only. All participants in the cohort consent at the outset to provide data to look at the benefit of the app self-help for well-being and mental health outcomes. Thus the three trial arms are: (1) usual practice self-monitoring control in which participants continue within the cohort with repeated assessment and using an app configured only for self-monitoring; (2) digital CBT self-help (active treatment control) in which participants receive the usual practice self-monitoring control and in addition are offered generic cognitive-behavioural self-help strategies within the app; (3) a personalised digital EC self-help in which participants receive the usual practice self-monitoring control and in addition are offered personalised digital self-help EC strategies within the app. Participants allocated to personalised digital self-help EC will receive two of four components corresponding to the two EC domains ranked lowest in their EC profile.

### Recruitment and study settings

**Fig. 1:**
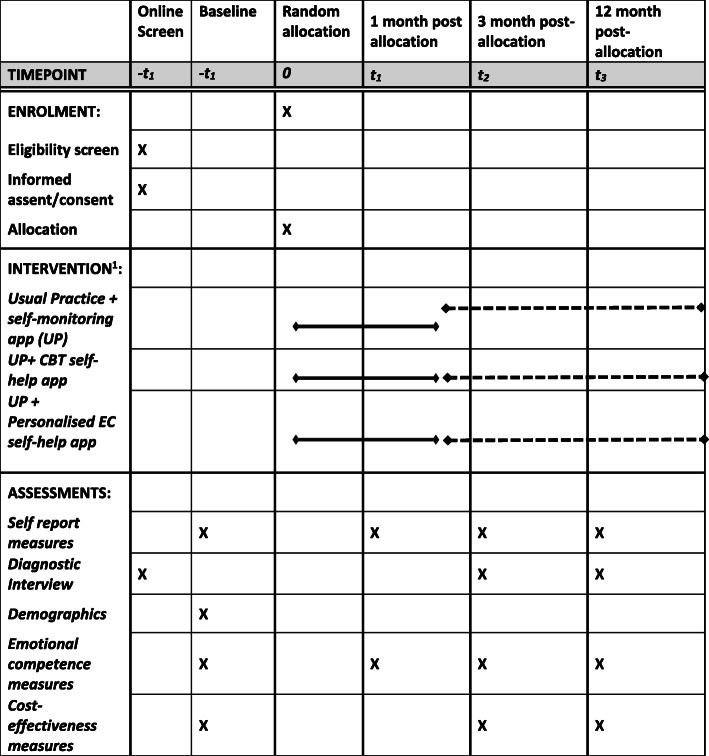
ECoWeB-PROMOTE and ECoWeB-PREVENT SPIRIT schedule of enrolment, interventions, and assessments

**Fig. 2:**
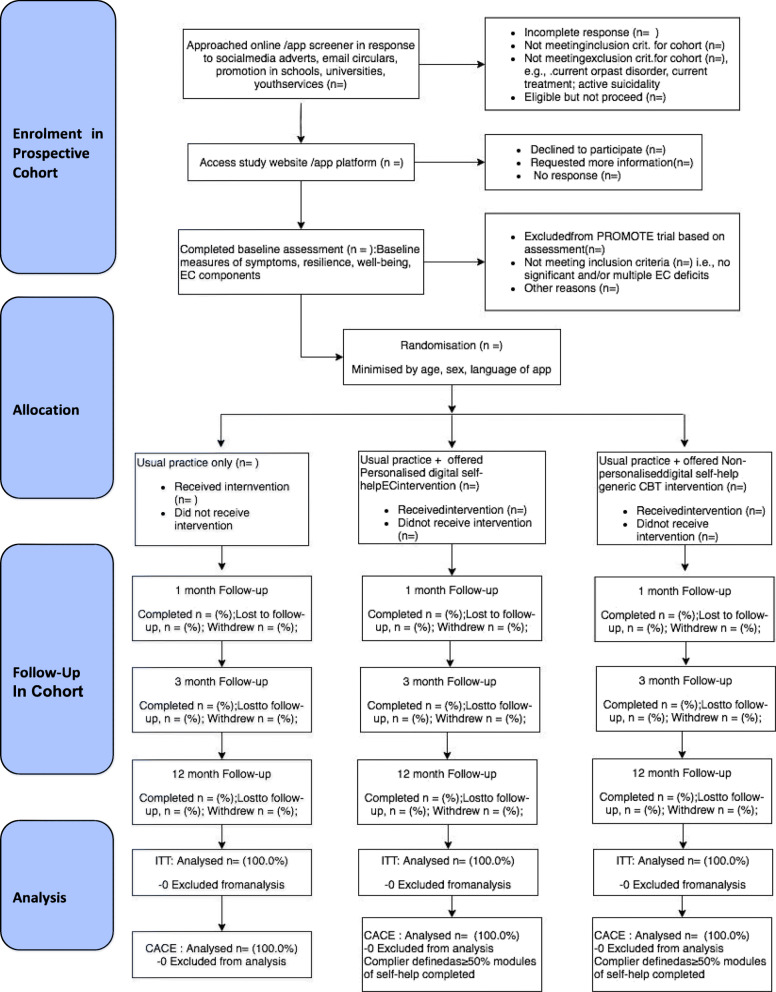
CONSORT flow diagram for ECoWeB-PROMOTE

**Fig. 3:**
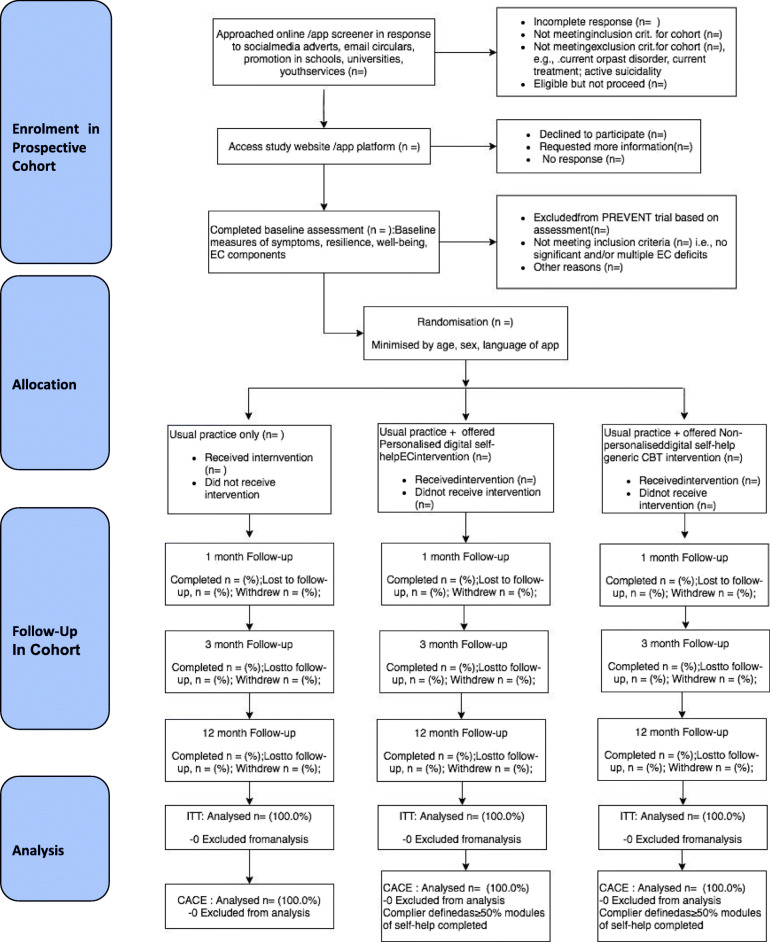
CONSORT flow diagram for ECoWeB-PREVENT

We seek to recruit 2142 participants across four different European countries (United Kingdom, Germany, Spain, Belgium) with central trial sites based at the University of Exeter, UK; LMU Munich, Germany; Universitat Jaume I, Castellon, Spain; and Gent University, Belgium (see Figs. 1, 2 and 3).

The recruitment strategy includes online and website advertising; email to mailing lists; newsletters and other circulars and noticeboards within willing schools, colleges and universities in the four countries. A social media campaign will be designed and prepared to be carried out on different social networks (e.g., Facebook, YouTube, Instagram, Twitter), including advertisements in social media; and blogs by YouTube influencers. It also includes mailings to members of relevant registers (e.g. city registers); recruitment via youth and mental health charities; brochures and posters in public areas; articles in local newspapers and press releases to the national press.

### Eligibility criteria

Eligible participants for the top-level cmRCT cohort will be: (1) young people aged 16 to 22 years old, (2) based in the UK, Spain, Germany, or Belgium (3) having basic literacy in at least one of English, Spanish, German, or Dutch, (4) able to provide informed consent and obtain parental consent for those aged under 18 years old in Germany and Belgium, and (5) having regular access to a smart phone (android or iOS) (see Table 1).

**Table 1:** Inclusion and exclusion criteria

**Inclusion criteria**	
16 to 22 years old	
Living in UK, Spain, Germany, Belgium	
Fluent in at least one of English, Spanish, German, Dutch (Flemish)	
Able to provide informed consent or parents willing to provide consent if under 18 years of age in Germany and Belgium	
Regular access to Android or iOS smartphone	
**Exclusion criteria**	
Current or Lifetime diagnosis of Major depressive Disorder (DSM-IV criteria, LIDAS self-report).	
Current use of antidepressant drugs or psychological interventions	
History of psychosis, bipolar disorder, substance dependence or other severe psychiatric disorder or current suicidality	

These trials are a primary prevention trial (PREVENT) and well-being promotion trial (PROMOTE), hence participants will be excluded from the cmRCT cohort at baseline if presenting with a current episode, or if they have a past episode of major depressive disorder (according to psychiatric DSM-IV criteria). This is determined in structured self-report electronic screening using the LIDAS instrument [64]. Other exclusion criteria include: active suicidality; any self-reported history of severe mental health problems such as bipolar disorder and psychosis; and currently receiving psychological therapy, counselling or psychiatric medication including antidepressants.

Specific inclusion/exclusion criteria will determine whether an individual is eligible for ECoWeB-PROMOTE or ECoWeB-PREVENT: elevated hypothesized vulnerability on EC profile based on the baseline assessment of EC skills is an inclusion criterion for ECoWeB-PREVENT and an exclusion criterion for ECoWeB-PROMOTE. Elevated vulnerability for a specified EC will be defined as scoring in the worst performing quarter on at least one measure assessing the component, and scoring in the worst performing third on the second measure for the same component (if two measures are being used for that component). This threshold is based on prior studies finding that individuals scoring in the worst quarter on measures of EC (e.g., rumination, appraisals, interpersonal vulnerabilities) have elevated risk for subsequent anxiety and depression [57, 65]. The exact quartile/tertile thresholds have been set following validation studies of the instruments in the target sample (16–22 year olds) across the four recruiting countries.

### Screening and consent procedure

Potential participants who are interested in the study are directed to our study website, (www.mymoodcoach.com) which provides further information, including eligibility criteria, and a pre-screener to check age and country. If appropriate, the website visitor is provided with the study information sheet and an initial consent screen to provide contact details (email; mobile phone number), and to provide informed consent to complete the baseline questionnaires. Individuals who are not suitable at pre-screen (e.g., outside of age range) will automatically be directed to a webpage explaining why they are not suitable for the trial. Those reporting mental health difficulties will be automatically guided to webpages providing information, guidance including to consult with their general practitioner (or equivalent), and weblinks and telephone numbers for help and support specific to each country, including contact details for the trial team.

After pre-screening, potential participants providing their email address are automatically emailed a copy of the information sheet, privacy policy and consent form so they can review them prior to giving any consent. Those potential participants who require consent from a parent or guardian (under age 18 in Germany or Belgium) are provided with a link to pass to their parent or guardian, who can then also view the information sheet and provide consent. On completion of parental consent, participants are automatically sent a link so that they can return to the assessment website, give their own consent and continue with the screening. Those not needing parental consent can consent straight away. Once this initial consent is provided, the participant proceeds to the baseline assessment.

Those meeting eligibility criteria following the baseline assessment are then asked to consent to take part in the prospective cohort and cmRCT, using an electronic information sheet, consent form and electronic signature. Once eligible participants consent to participate in the cmRCT, they will be deterministically allocated to the relevant trial and then randomised into one of the three conditions by a computer system. Consenting participants will be automatically signed up to use the relevant variant of the app via an application programming interface (API). The participant’s email address is used for app set-up.

### Baseline and follow-up assessments

The baseline assessment takes place after initial electronic informed consent is provided, and consists of web-based self-report measures to assess current and lifetime history of depression, current well-being, symptoms of anxiety and depression, social and work functioning, health-related quality of life, stressful events, and EC skills in each of the four study languages (English, German, Spanish, Dutch) (see outcome measures and Table 2). All of the questionnaires have been validated in all four languages. The Lifetime Depression Assessment Self-report questionnaire (LIDAS) [64] is used to assess lifetime major depression (MDD) diagnosis according to DSM criteria, and is largely based on the widely used Composite International Diagnostic Interview (CIDI). It has been proven to be effective for determining history of depression through self-report in an online digital format, matching the needs for the current study [64]. It consists of a conditional sequence of pre-programmed questions assessing all the diagnostic criteria for depression, with logic cut-outs so that subsequent questions are determined by prior questions, keeping the assessment brief.

**Table 2:** Measurements and Endpoints

			Follow-up (months)		
Web Assessment		Baseline	1	3	12
**Pre-screening**	Language, date of birth, country under which participating, self-reported mental health	✓			
**Informed Consent**		✓			
**Socio-demographics**	Age, sex, education, parental occupational status, country of birth, ethnicity	✓			
**Well-being**	WEMWBS questionnaire; primary outcome for PROMOTE	✓	✓	✓	✓
**LIDAS**	Self-report assessment of current and past MDE according to the definitions/ criteria of DSM-IV	✓		✓	✓
**Depression**	PHQ-9 questionnaire; primary outcome for PREVENT	✓	✓	✓	✓
**Anxiety**	GAD-7 questionnaire; secondary outcome	✓	✓	✓	✓
**General functioning**	Work and social adjustment (WSAS) questionnaire	✓	✓	✓	✓
**Quality of Life**	EQ 5D-3L measure	✓		✓	✓
**Estimate of health costs**	Adapted AD-SUS	✓		✓	✓
**Stress and adverse events**	Adverse Events Questionnaire, designed to assess common stressors in young people			✓	✓
**Emotional Competence – Emotional Regulation**	*Rumination using brooding subscale*^*a*^ *of Ruminative Response Scale of Response Styles Questionnaire; worry, using short Penn State Worry Questionnaire*^*a*^	✓	✓	✓	✓
**Emotional Competence – Emotional Knowledge and Perception**	Abbreviated Geneva Emotion Recognition Test (GERT)^a^ – performance measure of ability to recognise emotions from non-verbal cues; Components of Emotional Understanding Task (CEUT)^a^.	✓	✓	✓	✓
**Emotional Competence – Production (Social Appraisal)**	*Rejection Sensitivity Questionnaire*^*a*^ *to assess tendency for negative expectations of social situations*	✓	✓	✓	✓
**Emotional Competence – Production (Achievement Appraisal)**	Abbreviated Control Belief Scale; abbreviated; *abbreviated Perceived Academic Control scale*^*a*^; abbreviated Growth Mindset Scale and *Academic Value scales*^*a*^; abbreviated abbreviated Achievement Emotions Questionnaire	✓	✓	✓	✓

Note: Measures in italics are those used for selecting to ECoWeB-PREVENT vs ECoWeB-PROMOTE; measures indicated with ^a^ are those used in personalising intervention in EC self-help arm

All participants are entered into the prospective cohort and followed up electronically at 1, 3 and 12 months post-randomisation. At each follow-up point, participants will be automatically sent emails with links to enter their data into the assessment website. Each assessment point will involve an automated weekly follow-up and then email, text and telephone follow-ups by researchers to participants who haven’t yet completed the website assessment at 3 months and 12 months. Figure 1 and Table 2 give an overview of all measurements. Site researchers will be blind to treatment allocation, but will be available to participants to follow up risk and answer technical queries. Participants receive honorariums for the completion of each follow-up assessment.

### Randomisation, intervention delivery and masking

Participants will be randomised (in a 1:1:1 ratio) to the three intervention arms within each trial. Randomisation will be conducted automatically by means of a custom-built secure web service created and managed by the Exeter Clinical Trials Unit (ExeCTU), which interfaces with the trial database. To promote balance across key participant characteristics across intervention arms, randomisation will be minimised according to recruitment country (UK, Spain, Germany, Belgium), age (under 18 years old versus 18 years or older), and gender (male, female, both, neither). The minimisation algorithm will retain a stochastic element and the first 50 participants (PREVENT) and 150 (PROMOTE) will be allocated to their intervention arm by simple random allocation.

All of the online recruitment and randomisation will be automated and independent of trial researchers. The ExeCTU database will automatically send Monsenso (the app developer) the email address of the participant, their language and the version of the app that the participant is randomised to (control, active control or personalised EC version), enabling the participant to be set up with an account on the app. The online randomisation procedure will generate a unique trial identification code that is linked to the account on the app. All assessments will be routinely collected online using the assessment website following automated reminders, without the involvement of researchers. Site researchers will be blind to treatment allocation. Site researchers will prompt all participants to complete follow ups by phone, text and email if they do not respond to the automated reminders. Any unblinding in contact with a site researcher would be logged as protocol violations and only a researcher that remained blind will be able to prompt future follow-up from that participant.

### Interventions

#### Usual practice self-monitoring control

This control is carrying on in the prospective cohort, that is, usual practice plus the default basic version of the app featuring self-monitoring and the regular assessments within the cohort. Self-monitoring will include daily mood ratings and an ecological momentary assessment option (MoodTracker) for more detailed analysis of mood, activity and situational context. This self-monitoring is intended to help young people learn more about their emotional experiences and what influences them. Usual practice, as received by the young person outside of the trial, may include no provision of intervention, local provision of intervention, support from their GP/family doctor, local health services or youth services, or provision of intervention within their educational institution (e.g., well-being service at university; support and welfare staff at school). The nature of usual practice will be monitored and assessed by questionnaires at each follow-up assessment, determining what treatment and services participants have received since the last assessment.

#### Non-personalised digital self-help using generic CBT principles (active control group)

The active control will be a non-personalised digital self-help based on generic principles derived from cognitive behaviour therapy (CBT) for promoting mental well-being and for preventing poor mental health. This self-help app will use well-established generic cognitive-behavioural principles, including tips, advice, strategies and psychoeducation including on behavioural activation to increase positive activities, problem-solving and spotting and challenging negative thoughts, proven in RCTs to reduce symptoms of depression and anxiety and to promote well-being in young adults via online delivery [7, 8]. It will be delivered via an app using the same features, menu and structure as the personalised EC self-help to make the interventions as similar and as consistent in delivery as possible and to enable the interventions to be matched for delivery and format.

#### Personalised digital EC self-help (experimental intervention group)

The experimental arm is Emotional Competence (EC) self-help incorporating mobile smartphone application (app) delivery. As described earlier, there are four potential components focusing on different EC component processes: (i) improving accurate and functional emotion production via enhancing appraisals of achievement situations emphasizing increased perceived control and realistic perceived value (Achievement Appraisal); (ii) improving accurate and functional emotion production via training positive interpretations of ambiguous social events (Social Appraisal); (ii) improving emotional regulation by reducing maladaptive worry and rumination and replacing with constructive alternatives and problem-solving (Rumination); and (iv) enhancing emotional knowledge and perception through psychoeducation and learning tasks (Emotion Knowledge). The personalised digital EC self-help provides psychoeducation, tips, advice, exercises and training for each individual on the two EC components deemed to be most appropriate based on the baseline assessment. The app will include text, pictures, audio-recordings, animations, audio-exercises to practice (e.g., self-compassion), questionnaires with tailored feedback, quizzes, and gamification (e.g., badges, rewards, feedback). It will feature a menu structure including a dashboard to monitor notifications and progress, an explore function to graph the self-monitoring responses made by the participant, Challenges that provide learning exercises, and Tools that are brief strategies that young people can use in the moment when they need them. Within the app to support enhancing emotion knowledge, an innovative feature will be explored involving voluntarily provided audio-recordings being used to provide estimated feedback on perceived emotional arousal and dominance dimensions by machine learning techniques.

The app will be designed for iOS and Android use. All versions of the EC personalized self-help app will include the default self-monitoring features (including a regular daily mood rating and ecological momentary assessment) and gamification. To increase adherence on the app, completion of self-monitoring, Challenges (learning exercises) and Tools (practice of strategies) are each gamified, with badges earnt for progress on each and with electronic vouchers earnt when groups of badges are completed.

In recognition that behavioural intervention technologies rapidly evolve over time and that the technological instantiation can require debugging, updates, and enhancements of user interface, we will explicitly adopt the Trial of Intervention Principles approach [66]. This approach distinguishes between the underlying principles and behavioural strategies of a psychological intervention (e.g., goal setting, habit change), which need to remain unchanged to maintain trial integrity and robustness, and the specific technological instantiation of these principles (e.g., specific user flow, user experience and design), which can potentially change without compromising trial integrity. This provides the possibility to update the app – to correct bugs, upgrade systems and user interface – as long as any changes remain consistent with our a priori operationalisation of treatment principles and subject to independent agreement from our independent External Advisory Board.

#### Intervention adherence

The use of the app will be assessed and recorded including number of times the app is used and the number of badges received in the gamification system. A minimum intervention dose for the EC and CBT self-help arms (treatment compliance) will be defined a priori, based on the principle that users will benefit from learning new ideas (completing Challenges) and practising new skills (completing Tools). All participants in the usual practice self-monitoring control are defined as receiving the minimum intervention dose.

### Outcomes

Outcomes will be assessed at baseline (pre-randomisation) and 1, 3, and 12 months post-randomisation.

### Primary outcome

The primary outcome measure for ECoWeB-PROMOTE will be the 14-item Warwick-Edinburgh Mental Well Being Scale (WEMWBS) [67, 68], a leading validated self-reported index of well-being with excellent psychometric properties, at 3-month follow-up (the primary endpoint).

The primary outcome measure for ECoWeB-PREVENT will be the state depression score of the participants on the Patient Health Questionnaire-9 (PHQ-9) [69], a well-validated measure of depression at 3-month follow-up (the primary endpoint).

### Secondary outcomes

Secondary outcomes include: PHQ-9 [69] (for ECoWeB-PROMOTE), WEMWBS [68] (for ECoWeB-PREVENT). The Generalized Anxiety Disorder-7 (GAD-7) questionnaire will be used to assess anxiety symptoms [70]. The Work and Social Adjustment Scale (WSAS) [71] will be used to measure functioning with respect to work/education, home management, social leisure, private leisure and close relationships each rated from 0 not at all impaired to 8 severely impaired. Health-related quality of life will be assessed by the EuroQol instrument EQ-5D-3L [72]. The Adult Service Use Schedule (ADSUS-adapted) [73, 74] will be adapted for adolescents and young adults and is a well-established measure within the health economics field used to index relevant health and social care costs for trial participants. The Adverse Events Questionnaire is a brief measure designed to assess stressful events in young people across the domains of academic study, relationships, other bad experiences, hassles, which is proven to predict subsequent depression [75].

### Emotional competence skills

Different Emotional Competence skills are assessed through a battery of well-validated questionnaires and tasks, adapted and shortened for web-use: a validation study across all four countries determined shortened versions that maintained good psychometric properties. EC skills are assessed to allocate individuals to ECoWeB-PROMOTE or ECoWeB-PREVENT trials, to inform personalisation of treatment where applicable, as a manipulation check that interventions influence the targeted EC, and as potential mediators of intervention effect.

*Emotion Knowledge and Perception*: a brief version of the Components of Emotional Understanding Task (CEUT) [76] is used to assess understanding of all five emotion components of the Componential Emotion Approach (appraisals, action tendencies, bodily reactions, expression, subjective feelings). Participants read a series of emotion eliciting situations that describe potential emotional components, which the participant then rates for likelihood. The CEUT has good reliability and convergent validity.

The brief version of the Geneva Emotion Recognition Test (GERT-S) [77] is used to assess emotion perception: it is a 20-item performance-based emotion recognition test in which participants view short video clips of actors expressing 14 different emotions and then report which emotion had been expressed.

*Emotion Production*: To assess different components of appraisals, we will use (a) a shortened Emotional Disposition (EmoDis) tool [78] which asks participants to imagine experiencing four standardized emotional scenarios and rate their appraisals on three dimensions and the strength of three emotions; (b) a 4-item version of the Control Belief Scale which measures beliefs about one’s control, power and personal efficacy [79]; (c) abbreviated versions of the Perceived Academic Control scale [80]; Growth Mindset Scale [81] and Academic Value scales [82] to assess appraisals in achievement settings and intrinsic, utility, and attainment value. We will also use an abbreviated Achievement Emotions Questionnaire [83] to assess common emotions (enjoyment, anxiety, hopelessness, boredom) in different achievement contexts (school, university, work); the Rejection Sensitivity Questionnaire (ARSQ) [84] to assess concerns and expectations of the likelihood of rejection versus acceptance in social scenarios.

*Emotion Regulation:* Indices of emotion regulation will focus on repetitive negative thought in the form of worry and rumination: (a) the 5-item brooding subscale of the Ruminative Response Scale [85], a well-established measure of unhelpful rumination, which predicts subsequent depression; (b) the Penn State Worry Questionnaire -Abbreviated (PSWQ-A) [86], a well-validated 8-item measure of trait tendency towards worry.

The following descriptive variables will be assessed only at baseline: country of residence, age, gender, educational level, family’s occupational status, country of birth.

### Sample size

Because ECoWeB-PROMOTE and ECoWeB-PREVENT have different primary outcomes, the sample size for each trial was calculated using the respective primary outcome based on a minimum clinically important difference (MCID). The primary outcome for ECoWeB- PROMOTE is WEMWBS, which has a recommended MCID of 3.0 units [87]. This is based on observed smallest differences in prior studies and the lowest value greater than the observed standard error of measurement [88]. It was also the observed difference between a minimal self-help online CBT treatment and waiting list control at the 12-week endpoint for adults [89]. A standard deviation (SD) of 11.3 units is the most conservative estimate [88]. Using these values, with 90% power and a statistical significance threshold of 0.05, the sample size required for a 2-arm comparison would be 300 participants per arm. Accounting for 40% attrition at 3-month follow-up (the primary follow-up timepoint), a total of 500 participants per arm are required. Therefore, across the three arms of the trial, 1500 participants are required.

The primary outcome for ECoWeB-PREVENT is PHQ-9, which has an established MCID of 2.59 and SD of 5.4 [90]. Using the same power requirements as for ECoWeB-PROMOTE, the sample size required per arm is 93 participants. Accounting for 40% attrition at 3-month follow-up, 155 participants per arm are required, producing a total of 465 participants across the three arms.

A top level cohort, comprising young adults eligible for either trial, and assuming 70% eligible for ECoWeB-PROMOTE versus 30% for ECoWeB-PREVENT, will require 2142 participants (i.e., to achieve 1500 participants for PROMOTE, estimate to recruit 642 participants for PREVENT). For all participants recruited within the first 2 months from start of recruitment, we will calculate loss to 3-month follow-up and should it exceed 40%, we will re-adjust the sample size accordingly. In the event of loss to follow-up being less than 40%, the sample size will not be reduced.

### Statistical analysis plan

The primary analyses will be intention-to-treat (ITT) analyses [91] (i.e. all participants will be included in the analyses according to their randomised allocation) and based on complete case outcome data. The primary inferential analyses will compare across trial arms for the primary and continuous secondary outcomes at 3-months follow up using linear or logistic regression models with adjustment for baseline score, age (as a continuous variable rather than the dichotomised minimisation variable), country, and any other participant characteristics observed at baseline to be unbalanced across treatment arms.

A number of secondary analyses will be undertaken:

1. Complier Average Causal Effect (CACE) analysis [92, 93] to provide an estimate of a treatment effect accounting for pre-specified per protocol adherence and compliance with the treatment, whilst retaining the benefits of randomisation. Such models will be performed for the continuous primary and secondary outcomes at 3- and 12-month follow-up using observed data only, and will include the covariates adjusted for in the linear regression models.
2. Repeated measures analyses (using a mixed effects linear regression model with a random effect on participant) will be used to compare for primary and secondary outcomes across all follow up points of 1, 3 and 12 months, including data from participants with observed data for at least one of the three follow-up timepoints. Analyses will be performed to compare both active interventions with usual care, and to compare the personalised active intervention with the standard active intervention. A fixed effect interaction between timepoint and trial arm will be used to evaluate differential treatment effects across timepoints. Adjustments for baseline covariates will be made as for the primary analysis regression models; baseline score will be included as a covariate. Binary outcome measures will be analysed using logistic regression models with adjustments for covariates as above.
3. The pattern of missing outcomes will be examined and multiple imputation will be used to impute primary and secondary continuous outcomes. Imputation models will be informed by treatment arm, baseline scores, other covariates to be included in the model, and other baseline characteristics found to predict outcome or propensity for missingness (logistic regression models will be used to investigate the associations between baseline characteristics and missingness). Results of imputed models will be compared to primary analysis complete case ITT models.

(4) Mediation analyses will be undertaken to gain insight into mechanisms that could explain the potential effect of the interventions on primary outcomes. We will use modern causal inference methods using structural equation modelling or parametric regression models to assess mediation effects [94] including through changes in the EC variables. In addition, we will investigate potential moderation of the interventions by site, age, and sex.

Analyses will be undertaken by a statistician blinded to group allocation and using Stata v.15.

### Health economic analysis plan

The economic analyses will use a cost-consequence approach [95] for each country in the study, using a 12 month time horizon. Health and social care utilisation will be identified and collected using an adapted version of the Adult Service Use Schedule (AD-SUS) questionnaire, which quantifies the use of healthcare resources, use of medication and employment and time off work over the trial including follow up [74]. Adaptation will include time away from school or college together with changes to the wording to ensure relevance across the four countries. Unit costs of health and social care will be taken from appropriate national publications to reflect differences in costs between countries. Productivity losses will be valued using the human capital approach [96]. Wage rates for each country will be derived from European sources such as Eurostat. Outcomes will be assessed using the EQ-5D-3L [72], which will be converted to utility values using the EuroQoL general population tariff values for each country. Analyses will be on the intention-to-treat basis, and will be based on observed data only. Within country analyses of the differences between the trial arms will be undertaken. Although the distribution of costs is commonly skewed in populations of this kind, analyses will compare mean costs between groups using standard parametric regression models adjusted for minimisation variables and baseline costs. The robustness of the parametric tests will be confirmed using bias-corrected, non-parametric bootstrapping [97, 98]. As with primary and secondary outcomes, between group differences in costs and EQ-5D-3L will be presented as means and 95% CIs using Stata v15. Given the differences in cost structures (health and social care, and wage rates) and differences in the population tariffs between countries, no formal analyses of differences between costs and utility values between countries will be undertaken. However, the differences between the number and type of resource use and days away from education or work and employment will be examined.

### Qualitative analyses

During the cmRCT, implementation science approaches will examine the feasibility, fidelity, and acceptability of the interventions and examine the sustainability of uptake, pathways to increased uptake and usage, and potential barriers and obstacles to implementation. This process evaluation will identify enablers and barriers to the EC approach and conditions for implementation. The Consolidated Framework for Implementation Research (CFIR) [99] model will be used to examine the key domains of relevance and mediators and moderators for the implementation of the EC and CBT self-help apps. We will use a mixed methods approach, with quantitative and qualitative methods [100] combined with pragmatic measures, consistent with the MRC framework for developing and evaluating complex interventions [101, 102] and with constructs incorporating Theory of Change approaches [103]. Thirty to forty participants will be interviewed immediately post-intervention and at their 3 month follow-up, matching to their quantitative data collection points to reduce Hawthorne effects and to facilitate data integration at the analysis stage. We will ask about the following implementation outcomes for personalized EC and control interventions: Feasibility (e.g., how easy/difficult is it for young people to do this intervention; do they complete it?); Fidelity (e.g. how consistently is it experienced across the different settings?); Penetration (e.g., how well has it reached those young people for whom it has been primarily designed?); Acceptability (e.g., do young people like to use it? Is it more acceptable in certain cultural groups?); Sustainability (e.g., How well can it continue to be made available to relevant young people?); Cost (e.g., what is the intervention’s potential economic impact on downstream health service use, educational attainment and employment). Exact subgroups will be informed by the development phase and early app usage statistics during the trials.

### Organization, quality assurance and data management

Research data will be automatically collected in a pseudonymised manner through an electronic data capture system delivered from the website through to the central study database. In the first instance all participants will be directed to the website to provide their data. Questionnaires from respondents who prefer a paper version or who respond to follow-ups by telephone will be entered into the system by a site researcher research assistant and this variation in data collection method will be recorded. All data will be kept securely and confidentially and only accessed by specified researchers in each trial site. The central data-management team will use de-identified backups for the monitoring of the overall progress and data quality. Ultimately, a comprehensive de-identified dataset will be produced that includes data from all four research sites.

### Trial status

The Trial was registered in ClinicalTrials.gov. Number of identification: NCT04148508 (www.clinicaltrials.org). November 2019. Recruitment will commence in September 2020.

## Discussion

In recent years, improving the mental health of young people has been identified as a global health priority [4, 104]. Improving the mental health of young people includes both the prevention of poor mental health, such as the onset of anxiety and depression, and the promotion of increased well-being. Effective approaches to improve prevention and well-being promotion need to be widely accessible and highly scalable so that they can be reach large numbers of young people. One potential approach to delivering a scalable intervention is through the use of mobile apps (m-health) as the majority of young people regularly use mobile devices.

Whilst there is emergent evidence that mental health apps can be beneficial, to date the majority of apps available have not been tested in controlled trials and those that have been tested have been examined in relatively small samples. Large-scale intervention studies to establish the true impact of m-health apps on the promotion of well-being and the prevention of poor mental health are therefore needed specific to this age group.

The ECoWeB trial is an important contribution to this field by aiming to deliver one of the largest randomized controlled trials (*n* = 2142) to specifically examine the efficacy of m-health apps on the promotion of well-being and the prevention of poor mental health in 16–22 year olds across four European countries. More specifically, the study evaluates the efficacy of two different m-health strategies – a personalised self-help EC training smartphone app versus a generic CBT self-help smartphone app. As such, the ECoWeB trial will contribute to our knowledge of the efficacy and acceptability of different self-help mobile apps to improve mental health in young people. Moreover, ECoWeB is one of the first mental health trials to incorporate a personalization element as a test of whether a personalised approach can enhance outcomes relative to established generic interventions. In the personalised self-help EC training condition, participants will receive components matched to their respective abilities in emotional competence skills, to test if such matching improves treatment efficacy and usage compared to a standard non-matched intervention.

Because the trial is conducted across four distinct European states (UK, Germany, Spain, Belgium), it is envisaged that any findings could be generalised more broadly for young people across Europe and potentially around the world. By including repeated measurement of EC skills, mental well-being, and symptoms of anxiety and depression across the longitudinal cohort of the study, we also hope to learn more about the relative interaction of EC skills, well-being and mental health. For example, the trial design will enable us to determine which m-health strategies do improve EC skills, and whether this improvement in EC skills in turn predicts improvements in well-being. We hope that the lessons gained from the trial will enable the further development and enhancement of evidence-based m-health self-help strategies to promote well-being and prevent poor mental health in young people, and thereby contribute to addressing the priority of improving mental health in young people.

## Data Availability

Anonymised datasets arising from this trial will be made available after the primary outcomes are published to researchers and other groups via request to a data committee within the Consortium via the University of Exeter’s open access data system Open Research Exeter (ORE). ECoWeB partners will have access to the final trial dataset, commensurate with the grant Consortium Agreement. The results will additionally be updated on ClinicalTrials.gov Identifier: NCT04148508. The ECoWeB consortium plans to communicate trial results through peer-reviewed open access publications and direct reports to TSC, sponsor, and participants.

## Abbreviations

CACE: Complier average causal effect
DSM-IV: Diagnostic and Statistical Manual of Mental Disorders, Fourth Edition
GAD-7: Generalized Anxiety Disorder-7
ITT: Intention-to-treat
MDD: Major depressive disorder
MI: Multiple imputations
NIHR: National Institute for Health Research
NRES: NHS National Research Ethics Service
PHQ-9: Patient Health Questionnaire
RCT: Research control trial
TSC: Trial Steering Committee
